# Octreotide and celecoxib synergistically encapsulate VX2 hepatic allografts following transcatheter arterial embolisation

**DOI:** 10.3892/etm.2013.897

**Published:** 2013-01-16

**Authors:** HUAN TONG, XIAO LI, CHUN-LE ZHANG, JIN-HANG GAO, SHI-LEI WEN, ZHI-YIN HUANG, CHENG-WEI TANG

**Affiliations:** 1 Department of Gastroenterology, State Key Laboratory of Biotherapy, West China Hospital, Sichuan University, Chengdu 610041, P.R. China; 2 Division of Peptides Related with Human Diseases, State Key Laboratory of Biotherapy, West China Hospital, Sichuan University, Chengdu 610041, P.R. China

**Keywords:** hepatocellular carcinoma, tumour encapsulation, clear cell, angiogenesis, transcatheter arterial embolisation, octreotide, celecoxib

## Abstract

To evaluate the encapsulation of VX2 hepatic allografts in rabbits induced by octreotide and celecoxib administration following transcatheter arterial embolisation (TAE), rabbits with hepatic VX2 allografts were divided into four groups: control, TAE, octreotide + celecoxib (O+C) and the multimodality therapy (TAE+O+C). Allograft metastasis, capsule thickness and percentage of clear cells were measured and vascular endothelial growth factor (VEGF) and CD31 were detected by immunohistochemistry and reverse transcription-polymerase chain reaction (RT-PCR) analysis. The extrahepatic metastases of each intervention group were significantly fewer than those of the control group, with the TAE+O+C group exhibiting the fewest extrahepatic metastases. The TAE+O+C group had the greatest proportion of clear cells and thickest capsule on day 30. Increased capsule thickness was negatively correlated with tumour metastasis. In addition, VEGF expression levels assessed by immunohistochemistry and RT-PCR in the three intervention groups were significantly lower than those in the control group. Furthermore, the TAE+O+C group had a significantly reduced CD31 count induced by TAE. These results demonstrate that TAE, followed by long-term administration of octreotide and celecoxib, synergistically inhibits VX2 hepatic allograft metastasis by increasing the proportion of clear cells, promoting encapsulation and inhibiting angiogenesis.

## Introduction

The efficacy of therapies for hepatocellular carcinoma (HCC) is poor. Curative therapies, including resection, liver transplantation or percutaneous treatments benefit only 30% of patients (1). Even so, the majority of surgically treated patients show recurrence within 5 years of resection and this is linked to the high mortality of patients with resected HCC (2). Patients with large and multiple lesions exceeding the Milan criteria have been widely treated by transcatheter arterial embolisation (TAE) due to its precisely targeted, minimally invasive, repeatable and well-tolerated method. Although occlusion of tumour-feeding arteries may lead to extensive necrosis in vascularised HCC, hypoxia and ischemia of tumour tissue may produce large quantities of factors capable of inducing significant angiogenesis in the residual viable tumour, promoting recurrence and metastasis and consequently counteracting the efficacy of TAE (3,4).

Peri-procedural use of anti-angiogenic agents is recommended in order to overcome the disadvantages of TAE. However, the efficacy of those agents remains uncertain (5–7). The upregulation of cyclooxygenase-2 (COX-2), a key enzyme in arachidonic acid metabolism, is believed to be involved in hepatocarcinogenesis (8,9) and induce HCC angiogenesis via vascular endothelial growth factor (VEGF) (10,11), making COX-2 a rational therapeutic target for selective COX-2 inhibitors, including celecoxib. Somatostatin (SST) is one of the regulatory peptides for arresting the growth of HCC and the overexpression of SST receptors has also been identified in HCC (12). Our previous studies demonstrated that a combination of a COX-2 inhibitor with an SST analogue not only had an enhanced anti-proliferative effect and suppressed the metastasis of HCC in nude mice (13) but also prolonged the survival of rabbits with liver cancer that received TAE (14).

Various histopathological factors, including tumour size, tumour number, vascular invasion and tumour encapsulation, have been reported to be related to the prognosis of HCC. One study indicated that encapsulation is a favourable factor in large HCCs (>5 cm) and that encapsulation may act as a barrier to prevent the spread of tumour cells (15). However, few antitumour regimes stimulate the encapsulation of HCC. The current study aimed to evaluate the encapsulation of VX2 hepatic allografts in rabbits induced by octreotide and celecoxib administration following TAE.

## Materials and methods

### Animal experiments

All animal experiments were approved by the Institutional Animal Care and Use Committee of Sichuan University and were conducted according to local laws set by Sichuan University. Adult New Zealand White male rabbits weighing 2.3–2.5 kg were purchased from the Experimental Animal Centre of West China Medical Centre, Sichuan University. VX2 allograft-bearing rabbits were purchased from the Union Hospital, Huazhong University of Science and Technology (Wuhan, China).

The establishment of VX2 hepatic allografts in rabbits, TAE procedure and experimental grouping were the same as in our previous study (14). Briefly, 72 rabbits were randomly assigned into four groups. The VX2 tumours were orthotopically implanted into the livers of the rabbits. A total of 67 VX2 allograft-recipient rabbits were divided into 4 groups and treated as follows: i) control (n=18), the sham-operated animals received normal saline (NS) daily, intragastrically and subcutaneously; ii) TAE (n=17), the animals received the TAE procedure and then NS in the same way as the control group; iii) octreotide + celecoxib (O+C; n=16), the animals received sham surgery and then subcutaneous administration of octreotide (Novartis Diagnostics, Emeryville, CA, USA) at a dose of 37 *μ*g/kg/day plus intragastric administration of celecoxib (Pfizer, New York, NY, USA) at a dose of 22.2 mg/kg/day and iv) multimodality therapy (TAE+O+C; n=16), the animals received TAE followed by subcutaneous administration of octreotide at a dose of 37 *μ*g/kg/day plus intragastric administration of celecoxib at a dose of 22.2 mg/kg/day. Eight animals of each group were sacrificed after 30 days of treatment. The other animals were raised until spontaneous mortality or were sacrificed after 80 days of treatment.

Once the tumours were removed and weighed, metastatic foci were carefully searched for in organs. The tumour inhibition rate (%) = [(tumour weight of control − tumour weight of treatment group)/tumour weight of control] × 100. Tumour tissue was fixed in neutral buffered formalin for histological examination or 4% glutaraldehyde for transmission electron microscopy, or stored in −80°C ultra-low freezer for reverse transcription-polymerase chain reaction (RT-PCR).

### Morphological evaluation of VX2 allografts in rabbits

Paraffin-embedded specimens were sliced into 5-*μ*m sections and stained with haematoxylin and eosin (H&E) for histological evaluation in a single-blinded fashion. Clear cells in the VX2 allografts, due to cytoplasmic accumulation of glycogen and fat droplets that dissolved during the H&E staining process and left behind a ‘clear’ cytoplasm, were detected and counted in each tumour allograft (16). Encapsulation of the tumours was evaluated and the capsule thickness of complete capsules was measured in pixel pitches using Image-Pro Plus 6.0 software (Media Cybernetics, Rockville, MD, USA) and then was normalised into *μ*m. Each value was the mean of five visual fields in which duplicate measurements were made.

Tumour specimens from each group were also immersed in 4% glutaraldehyde (pH 7.4) at 4°C for 24 h, postfixed in 1% osmium tetroxide for 1 h and embedded in Epon 812 following dehydration. Following double staining with uranyl acetate and lead citrate, ultrathin sections (60 nm) were examined with a transmission electron microscope (H-600IV, Hitachi, Tokyo, Japan).

### Immunohistochemistry for VEGF and CD31

Immunohistochemistry was performed on 5-*μ*m paraffin-embedded tissue sections on poly-L-lysine coated glass slides. The sections were deparaffinised and treated with microwaves for 15 min. For non-specific blocking, 10% goat serum was added and incubated for 20 min at room temperature. Then the VEGF antibody (ab288775; Abcam, Cambridge, MA, USA) and the CD31 antibody (08-1425; Zymed Laboratories Inc., San Francisco, CA, USA) at a 1:250 dilution were added to the individual sections. Positive reactions were revealed by the streptavidin-biotin-peroxidase technique. Sections were incubated with 3,3′-diaminobenzidine (0.05% 3,3′-diaminobenzidine in 0.05 M Tris buffer, pH 7.6 and 0.01% hydrogen peroxide) and counterstained with Mayer’s haematoxylin. Image-Pro Plus 6.0 software was used to score the integrated optical density (IOD) from the VEGF expression in the tumour cells and count the number of CD31 per visual field (magnification, ×200) in a single-blinded fashion. Each value was the mean of five visual fields in which duplicate measurements were made.

### RT-PCR for VEGF analysis

Total RNA was extracted from allograft tissue using the TRIzol reagent (15596-026; Invitrogen Life Technologies, Carlsbad, CA, USA). Quantification and purity of extracted RNA were determined by the ratio of absorbance at 260 and 280 nm (A260/A280) with a spectrophotometer (GeneQuant 1300; Biochrom, Holliston, MA, USA). Reverse transcription and PCR amplification were conducted using a thermal cycler (PTC-100; Bio-Rad, Hercules, CA, USA), in accordance with the instructions of the RT-PCR core kit (K1622; Fermentas, Hanover, MD, USA). The primer sequences for the sense and antisense chains were as follows: glyceraldehyde 3-phosphate dehydrogenase (GAPDH; XM_002714697): 5′-TCT CGT CCT CCT CTG GTG CTC T-3′ and 5′-AAG TGG GGT GAT GCT GGT GC-3′; and VEGF (NM_001082253): 5′-ATG GCA GAA GAA GGA GAC-3′ and 5′-ATT TGT TGT GCT GTA GGA AG-3′. The PCR cycle profile was 94°C for 30 sec, 52°C for 60 sec and 72°C for 60 sec, for 30 cycles. The amplification was terminated by a final extension step at 72°C for 2 min. A positive control (kidney RNA) and an internal control (GAPDH) were amplified at the same time. PCR products were quantified using a gel membrane, which was scanned into an imaging system (Gel Doc 2000, Bio-Rad). The data were normalised as a ratio of gray scale (IOD) of objective band over GAPDH.

### Statistical analysis

Quantitative data are expressed as the mean ± standard deviation and tested by one-way analysis of variance (ANOVA). Qualitative data were tested by the Chi-square test and correlation analysis was also conducted to verify the correlation between two parameters. Statistical analysis was performed using SPSS 13.0 for Windows (IBM, New York, NY, USA). P<0.05 was considered to indicate a statistically significant difference.

## Results

### Effect of O+C treatment on extrahepatic metastases following TAE

**Figure 1.**
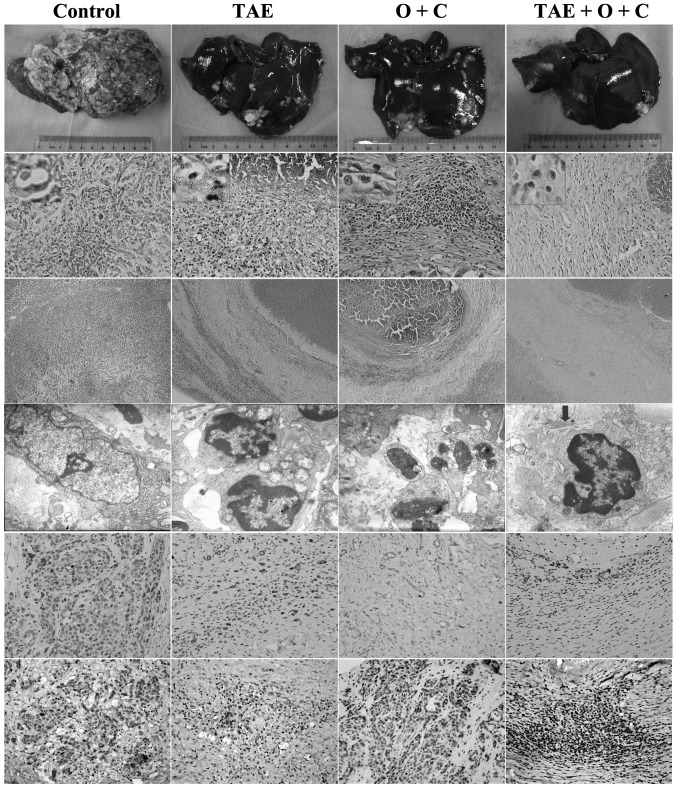
VX2 allografts of the four groups on day 30. Row 1, gross morphology of allografts; row 2, clear cells (haematoxylin and eosin staining; magnification, ×400 and ×100); row 3, capsules (haematoxylin and eosin staining; magnification, ×100); row 4, ultra-structure of the tumour cells. The arrow indicates a collagen bundle (transmission electron microscope; magnification, ×10,000); rows 5 and 6, positive expression of CD31 and vascular endothelial growth factor (VEGF) with brown grains (immunohistochemical staining; magnification, ×100). TAE, transcatheter arterial embolisation; O+C, octreotide + celecoxib.

The total intrahepatic foci weight of each intervention group was significantly lower than that of the control group on day 30 and during days 30–80 (Table I and Fig. 1, row 1). The inhibition rate of the TAE+O+C group was the greatest among the three intervention groups during the whole experiment (Table I). Extrahepatic metastasis was detected in the control group on day 30; however, it was greatly reduced in the three intervention groups (P=0.006; Table I). During days 30–80, extrahepatic metastasis in the three intervention groups remained significantly lower than that of the control group (P=0.007). Over this time period, the TAE+O+C group demonstrated the least extrahepatic metastasis among the three intervention groups.

### Effect of O+C treatment on clear cell number and capsule thickness following TAE

Microscopically, the nuclei of clear cells were mainly located centrally or slightly eccentrically with dense or occasionally clumpy chromatin. They were detectable in the VX2 hepatic allografts of the four groups (Fig. 1, row 2). The TAE+O+C group demonstrated the highest proportion of clear cells during the whole experiment (P<0.05; Table I).

Complete capsule morphology was displayed in only half of the allografts in the control group on day 30; however, they were formed in the allografts in the majority of the TAE, O+C and TAE+O+C group animals (Table I). Forty percent of the allografts in the control group exhibited partial formation of capsules at spontaneous mortality. There were complete capsules around all the allografts in the three intervention groups on day 80. The three interventions significantly increased the thickness of the complete capsules during the whole experiment (P=0; Table I and Fig. 1, row 3). The thickest capsules, ∼5.4 times the size of that in the control group, were observed in VX2 allografts treated with the TAE+O+C regime. The capsules were often adjacent to the necrotic tissues.

An irregular shape of the nuclear membrane and the nucleolus and extension of the endoplasmic reticulum were observed in the control group. Swelling mitochondria were displayed in the tumour cells of the TAE group. A greater number of apoptotic bodies in the cells of VX2 allografts were detected in the O+C group. Additionally, collagen bundles surrounding the tumour cells were clearly increased in the TAE+O+C group (Fig. 1, row 4).

**Figure 2.**
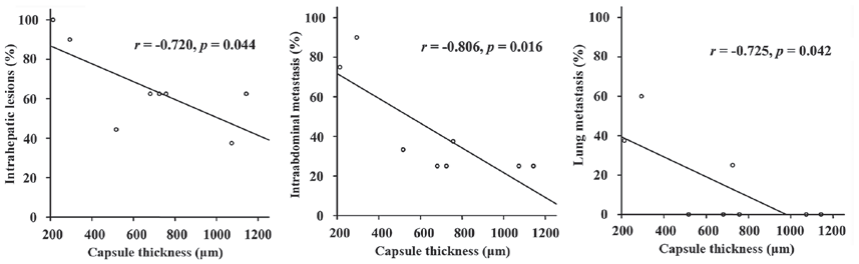
Correlations between capsule thickness and intrahepatic lesions, intra-abdominal metastasis and lung metastasis.

Compared with partial capsules, complete capsules greatly reduced the intrahepatic lesions and intra-abdominal metastasis, as well as lung metastasis (P<0.05; Table II). The thickness of the complete capsules was negatively correlated with the intrahepatic lesions and intra-abdominal and lung metastasis (P<0.05; Table II and Fig. 2).

### Effect of O+C treatment on VEGF expression following TAE-induced angiogenesis

**Figure 3.**
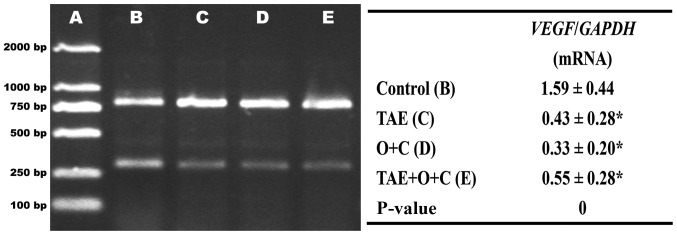
Expression of VEGF mRNA in VX2 allografts using RT-PCR. Lane A, marker DL-2000; lane B: control group; lane C: TAE group; lane D: O+C group and lane E: TAE+O+C group. mRNA products of VEGF were 250–500 bp and those of GAPDH were 750–1000 bp. Each value was the mean ± SD from 8 animals in each group in which duplicate measurements were made. ^*^P<0.05 vs. control. VEGF, vascular endothelial growth factor; RT-PCR, reverse transcription-polymerase chain reaction; TAE, transcatheter arterial embolisation; O+C, octreotide + celecoxib; GAPDH, glyceraldehyde 3-phosphate dehydrogenase; SD, standard deviation.

The capsules of the VX2 hepatic allografts were rich in microvessels (Fig. 1, row 2), which was revealed with the positive staining of CD31 (Table I and Fig. 1, row 5). Following the TAE procedure, angiogenesis in the capsules significantly increased (Table I and Fig. 1, row 5). However, the TAE+O+C regime significantly downregulated the expression of CD31 and VEGF in the VX2 allografts on day 30, compared with the TAE group (Table I and Fig. 1, rows 5 and 6). Although the expression of VEGF mRNA in the three intervention groups were significantly lower than that in the control group, there was no significant difference in the expression of VEGF mRNA between the TAE and TAE+O+C groups (Fig. 3).

## Discussion

Clear cell HCC has a particular histological type, and accounts for between 0.4 and 37% of all HCC cases (16–19). Patients with primary clear cell carcinoma of the liver often have a higher rate of HCV infection and capsule formation associated with suppressed vascular invasion. The prognosis is better than that of patients with common type HCC, which is related to the ratio of clear cells. There are few studies that demonstrate the effect of enhancing the proportion of clear cells in HCC. TAE followed by long-term administration of octreotide and celecoxib synergistically induces the secondary clear cells in HCC and therefore greatly prolongs the survival of rabbits with VX2 hepatic allografts (14).

Encapsulation, defined as the formation of a clear fibrous layer with collagen content, acts as a barrier to prevent the spread of tumour cells. Capsule formation has been observed in ∼90% of primary clear cell carcinomas of the liver (20,21). The TAE procedure significantly enhanced the completeness of the capsules when compared with controls. Furthermore, TAE+O+C combination therapy significantly enhanced capsule thickness resulting in increased number of clear cells in the VX2 hepatic allografts. Hepatic stellate cells (HSCs) are the major cellular component of the HCC capsule and the formation of a HCC capsule may start from the activation of HSCs (15). Encapsulation was usually related to ischemic necrosis of surrounding tissue that may increase production of extracellular matrix in VX2 hepatic allografts. The collagen bundles adjacent to the tumour cells may also induce apoptosis of the tumour cells.

One study considered that an early HCC tumour is an ill-defined nodule without fibrous capsule formation, the fibrous capsule appears as the tumour size increases and the survival of patients with encapsulated HCCs is poorer than that of patients with HCC without encapsulation (22). In contrast to this observation, the current study revealed that encapsulation of VX2 hepatic allografts was negatively related to tumour growth and metastasis. Moreover, there are a number of controversies on vascular invasion and capsule formation (15,23). The present study identified that the TAE+O+C regimen significantly inhibits TAE-induced angiogenesis and VEGF expression in the capsules, as well as increases the completeness and the thickness of the capsules. The main fibrogenic stimuli for stromal cells requires further elucidation.

Although encapsulation of HCC is considered as an important bio-behavior, which would be beneficial to the host, no regime has previously been reported to encapsulate HCC as demonstrated in this study. The potential inhibition of VX2 hepatic allograft growth and metastasis with the TAE+O+C regime relates to the increased proportion of clear cells, the encapsulation and anti-angiogenesis effects.

## Figures and Tables

**Table I.** Variation of VX2 allografts and their metastasis in each group.

	Control	TAE	O+C	TAE+O+C	P-value
Total animal number	18	17	16	16	-
Data on day 30					
Total intrahepatic lesions					
Weight (g)	63.8±57.8	8.2±7.0^a^	14.6±21.5^a^	5.7±4.9^a^	0.002
Inhibition rate (%)	-	87.1	77.1	91.1	-
Allografts					
Clear cells (%)	5.1±1.9^b^	6.6±3.6	5.7±2.5^b^	9.4±2.7	0.043
VEGF (IOD, ×10^5^)	3.43±2.01	1.05±0.44^a^	0.71±0.59^a^	0.44±0.30^a,c^	0
Capsules					
Partial/complete (n/n)	4/4	1/7^a^	0/8^a^	0/8^a^	0.017
Thickness (*μ*m)	213±59	681±290^a,b^	757±302^a,b^	1143±322^a^	0
CD31 (number/field)	22.5±6.1	38.6±4.6^a^	12.2±2.6^a,c^	11.0±2.2^a,c^	0
Extrahepatic metastasis (%)	100	25^a^	37.5^a^	25^a^	0.006
Data during days 30–80					
Total intrahepatic lesions					
Weight (g)	105.5±70.5	8.4±13.6^a^	19.6±20.8^a^	4.8±4.5^a^	0
Inhibition rate (%)	-	92.0	81.4	95.5	-
Allografts					
Clear cells (%)	3.2±2.8	7.6±4.1	8.3±5.9	12.3±5.2^a^	0.026
Capsules					
Partial/complete (n/n)	4/6	0/9^a^	0/8^a^	0/8^a^	0.010
Thickness (*μ*m)	294±130	517±235^b^	725±229^a,b^	1073±432^a^	0
Extrahepatic metastasis (%)	100	55.6^a^	37.5^a^	25^a^	0.007

P<0.05 vs.

a control,

b TAE+O+C,

c TAE. TAE, transcatheter arterial embolisation; O+C, octreotide+celecoxib; TAE+O+C, multimodality therapy; VEGF, vascular endothelial growth factor; IOD, integrated optical density.

**Table II.** Effects of capsules on tumour growth and metastasis.

	Intrahepatic lesions (%)	Intra-abdominal metastasis (%)	Lung metastasis (%)
Integrity of capsules			
Complete	65.5	36.2	8.6
Partial	100	88.9	66.7
P-value	0.035	0.003	0
Correlation with the thickness of capsules			
R-value	−0.720	−0.806	−0.725
P-value	0.044	0.016	0.042
